# Hospital Festivals and Meetings

**Published:** 1895-06-08

**Authors:** 


### HOSPITAL FESTIVALS AND MEETINGS.

### HOSPITAL FOR CONSUMPTION, BROMPTON.

The annual Court of Governors of the Brompton Hospital for Consumption was held last week, Mr. T. P. Beckwith in the chair, when the 54th annual report was adopted. The report showed that donations and legacies had been less in 1894 than in the previous year, but the number of annual subscriptions maintained. With regard to cases treated, the figures testified to a high average of attendances and admissions; 1,851 patients having been received into the wards, of which 1,277 were discharged, in many cases much benefited; 13,750 new out-patients had received treatment. In the course of the usual business the Earl of Derby was elected president of the hospital, vice the Duke of Portland. The committee of management was also re-elected, and the meeting closed with the customary votes of thanks.

### MIDDLESEX HOSPITAL.

The governors and committee of the Middlesex Hospital set an excellent example to the hospital world in their well attended quarterly courts and general meetings, and in the harmony and good fellowship which invariably distinguishes the administration of the affairs of this great institution. The court held on Thursday in last week was no exception to the rule. A goodly number of governors were present, including Sir Ralph Thompson, K.C.B., in the chair, Mr. Kegan Paul, Mr. Leopold Hudson, F.R.C.S., Canon Acheson, Colonel the Hon. C. G. Gathorne Hardy, Mr. Hoare (treasurer), and Dr. Sydney Coupland.

The report presented to the board stated that unfortunately the projected garden fête in aid of the new convalescent home in course of construction at Clacton-on-Sea would have to be postponed till next year, it being found impossible to secure the presence of any member of the Royal Family, and various changes in the medical staff and on the board were dealt with. In consideration of the long and valuable services of Miss Thorold, the lady superintendent, it was proposed, and unanimously agreed to, that her remuneration should be increased from £130 to £200 per annum. The new operating theatre was reported to be now complete and ready for use. Legacies of £1,000 from the Rev. W. Bindloss and £2,000 from the late Emily Jane Mullet were bequeathed to the hospital during the last quarter. The Chairman commented briefly on the various matters included in the report, which was then unanimously adopted *en bloc*; and the collector's report having been received, wherein it was shown that subscriptions had somewhat decreased, and the money from collecting-boxes—those inside giving a decrease and those outside a slight increase in amounts—having been announced, the court dissolved with the usual votes of thanks.

### EAST LONDON HOSPITAL FOR CHILDREN.

The Earl of Erroll presided at the festival dinner which was held on May 30th in the fine hall of the Company of Leathersellers in aid of the funds of the East London Hospital for Children. There was a large attendance, and the handsome sum of £2,400 was handed over to the secretary as the result of the after-dinner collection.

The first toast, that of "The Queen, Patron of the Hospital," was enthusiastically received. The Chairman, in proposing it, commented on the sympathy always shown by her Majesty to the poorest of her subjects in their sorrow and distress.

In proposing "Their Royal Highnesses the Prince and Princess of Wales and the Royal Family," the Chairman remarked that hardly a day passed without their names being associated with some scheme having for its object the welfare of the poor. H.R.H. the Duchess of Teck opened the new buildings of this hospital in 1877, and the Duke of Cambridge presided at one of the festival dinners.

After this toast had been duly honoured Miss Grainger Kerr sang "Spring is Here," one of Mrs. Edith Dick's charming compositions, which was highly appreciated.

The toast of the evening followed, "Success to the East London Hospital for Children." The Chairman, in giving it, expressed satisfaction at the presence of the ladies at this dinner. His own official connection with the hospital had not been limited to a cursory walk through the wards, or a perusal of a printed report, for he had been on the executive committee for many years, and could assert from personal knowledge that the administration was economical and effective. The children were treated with care and skill by the medical men, and they had most competent nurses. Therefore the claims of the hospital were not pressed with humility or apology, as good work should stand on its own merits. Stress was laid by the speaker on the disadvantages of the geographical position of the Children's Hospital, whilst the situation of the Great Ormond Street and Victoria Hospital was familiar to most people, thousands had never even heard of Shadwell. In proof of the appreciation enjoyed by the hospital in its own neighbourhood the Chairman related how the men had sent a message during a great coal strike to say that whatever happened the hospital should not run short of coal. In conclusion Lord Erroll appealed eloquently for comforts and luxuries for the children in hospital to equal those enjoyed by their own little ones. Mr. H. W. Trinder, in replying to the chairman, spoke of the starvation which aggravated the diseases of the poor, and especially of the children. He advocated the introduction of a crêche which he thought would be of extreme value in Shadwell. He also appealed for the help of ladies as assistants in the management of the hospital. Although they had occasionally asked the public for support they had never yet run into debt.

A song by Mr. Hirwen Jones and a violin solo by Miss Edith Robinson were followed by the toast of "The Board of Management," which Mr. F. Wootton Isaacson, M.P., said he had the greatest possible pleasure in proposing. Mr. C. A. Prescott replied, and Mr. George Russell gave "The Medical and Resident Staff," which Dr. T. H. Arnold Chaplin acknowledged. Other speeches followed, and cordial thanks were given to Lord Erroll for his kindness in presiding on the occasion. Amongst the guests present were the Earl and Countess of Arran, Mr. and Mrs. Arnold Trinder, Mr. H. W.

Trinder, Mr. F. Wootton Isaacson, M.P., the Rev. J. Kennedy, D.D., Mrs. Julian Sturge, Mr. Joseph Spence, Mr. Thomas Smith, Dr. Arnold Chaplin, Mr. and Mrs. Horace Cheston, Dr. Donbrin, Dr. Eustace Smith, Major Robert Carr, Mr. George Russell, Mr. Percy Phillips, and many others.

